# Concise Synthesis of (+)-β- and γ-Apopicropodophyllins, and Dehydrodesoxypodophyllotoxin

**DOI:** 10.3390/molecules23113037

**Published:** 2018-11-21

**Authors:** Jian Xiao, Guangming Nan, Ya-Wen Wang, Yu Peng

**Affiliations:** 1 University and College Key Lab of Natural Product Chemistry and Application in Xinjiang, Yili Normal University, Yining 835000, China; xiaoj2012@lzu.edu.cn (J.X.); nanguangming02@sohu.com (G.N.); 2 School of Life Science and Engineering, Southwest Jiaotong University, Chengdu 610031, China; ywwang@swjtu.edu.cn; 3 State Key Laboratory of Applied Organic Chemistry and College of Chemistry and Chemical Engineering, Lanzhou University, Lanzhou 730000, China

**Keywords:** aryldihydronaphthalene lignan, arylnaphthalene lignan, oxidation, synthesis

## Abstract

Herein, we present an expeditous synthesis of bioactive aryldihydronaphthalene lignans (+)-β- and γ-apopicropodophyllins, and arylnaphthalene lignan dehydrodesoxypodophyllotoxin. The key reaction is regiocontrolled oxidations of stereodivergent aryltetralin lactones, which were easily accessed from a nickel-catalyzed reductive cascade approach developed in our group.

## 1. Introduction

**Scheme 1:**
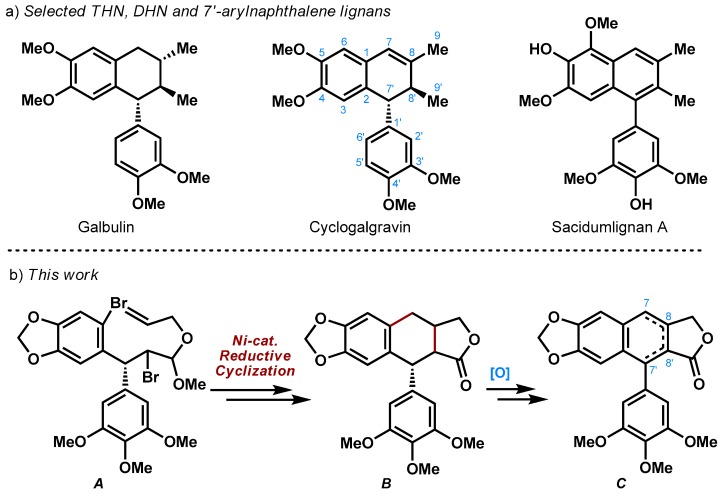
(**a**) Several arylnaphthalene lignans and their DHN and THN derivatives; (**b**) Our synthetic logic.

Lignans are a class of secondary metabolites in various plants, and most of them have demonstrated interesting biological properties [1,2], thus attracting the attention of the synthetic chemists [3,4]. Some of 2,7′-cyclolignans such as 7,8,8′,7′-tetrahydronaphthalene (THN), 7′,8′-dihydronaphthalene (DHN) and 7′-arylnaphthalene types are exemplified in Scheme 1a. Hong and co-workers used organocatalytic domino Michael–Michael–aldol reactions to construct THN skeleton of galbulin and realized its first enantioselective synthesis [5]. Barker and co-workers completed the first asymmetric synthesis of (−)-cyclogalgravin based on a key construction of C2–C7′ bond from in situ generated quinoid intermediate [6]. Notably, the other two structurally distinct class of lignans could also be obtained from a common precursor in their syntheses. Ramana et al. proposed a dehydrative cyclization of an aldehyde intermediate to build the DHN unit of sacidumlignan B, whose subsequent aromatization led to the synthesis of sacidumlignan A [7]. We were also involved in this fascinating field and achieved the synthesis of these three molecules through Ueno–Stork radical cyclization and Friedel–Crafts reaction [8,9]. However, almost all of the above syntheses applied stepwise strategies (i.e., a sequence of C2–C7′, C8–C8′, then C1–C7 bonds formation in our previous routes) for construction of the central core [10].

## 2. Results and Discussion

Recently, we completed a new synthesis of podophyllotoxin [11,12], an aryltetralin lignan used as building block for the chemotherapeutic drugs etoposide and teniposide. The key reaction is a Ni-catalyzed reductive tandem coupling [13,14,15,16,17,18,19] of dibromide ***A*** that led to the simultaneous construction of C8–C8′ and C1–C7 bonds in THN framework of B (Scheme 1b). We envision that this aryltetralin lactone could serve as an advanced intermediate for the unified synthesis of the titled arylnaphthalene, DHN and THN lignans ***C***, by means of the regioselective late-stage oxidation. Herein, we disclosed the preliminary results.

**Scheme 2:**
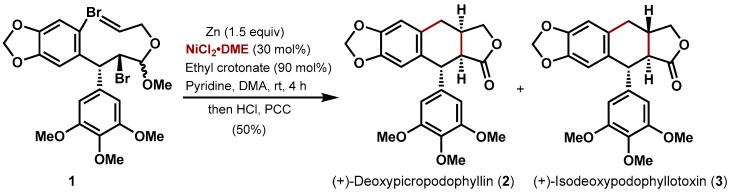
Reductive tandem cyclization for tetralin lactones.

Starting from the commercially available 6-bromopiperonal and 3,4,5-trimethoxyphenyl bromide, the chiral β-bromo acetal **1** was straightforwardly prepared as in gram-scale according to a known route [11]. Under a fully intramolecular reductive nickel-catalysis ligated by ethyl crotonate (Scheme 2), diastereodivergent (+)-deoxypicropodophyllin (**2**) and (+)-isodeoxypodophyllotoxin (**3**) were obtained in 50% overall yield after a conversion of acetal moiety to the corresponding lactone. With aryltetralin lactones **2** and **3** in hand, the designed regiocontrolled oxidation in central aliphatic ring could be executed (vide infra).

**Scheme 3:**
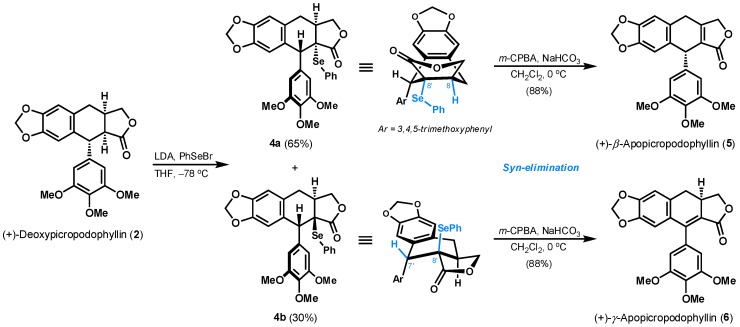
Regiodivergent oxidation of (+)-deoxypicropodophyllin (**2**).

**Figure 1:**
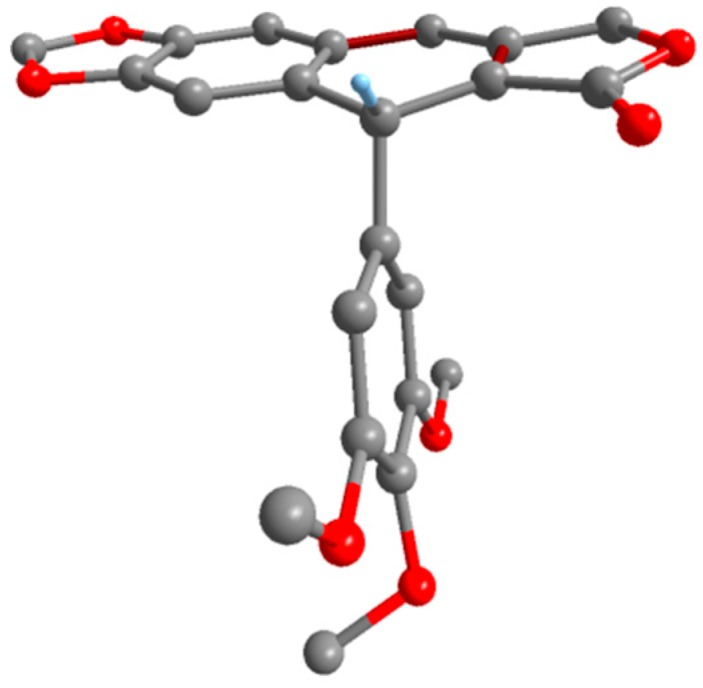
X-ray crystal structure of (+)-β-apopicropodophyllin (**5**), selected H atoms have been omitted for clarity.

First of all, the increase of an unsaturation degree at either C8–C8′ or C7′–C8′ location was pursued in order to get (+)-β-apopicropodophyllin (**5**) and (+)-γ-apopicropodophyllin (**6**) quickly. As shown in Scheme 3, the introduction of a phenylselenyl group at C8′ position of (+)-deoxypicropodophyllin (**2**) was done by an initial enolization and subsequent quench with phenylselenyl bromide (PhSeBr) at −78 °C. The generated products as two diastereoisomers (**4a** and **4b**) were separated by column chromatography on silica gel in 95% overall yield. The α-phenylselenide **4a** is supposed to adopt a pseudo-boat conformation, where the hydrogen atom at C8 is arranged *cis* to the -SePh. The requisite *syn*-elimination of phenylselenoxide in situ generated from oxidation of **4a** [20], eventually provided (+)-β-apopicropodophyllin (**5**) with in vivo insecticidal activity against the fifth-instar larvae of *Brontispa longissima* [21]. Its ^1^H NMR spectral data (Table S2) and optical rotation were in agreement with the reported data by Toste and Meyers [22,23]. The structure was later unambiguously confirmed by its single-crystal analysis (Figure 1) [24]. In contrast, the hydrogen atom at C7′ is oriented at *cis*-position of C8′-PhSe in the favored half-chair conformer of β-phenylselenide **4b**. Thus, a double bond within C7′–C8′ was formed upon the subjection of **4b** to *m*-CPBA, therefore affording to (+)-γ-apopicropodophyllin (**6**) in 88% yield. As shown in Table S3, ^1^H NMR spectra of the synthetic **6** was accord with the literature [25].

**Scheme 4:**
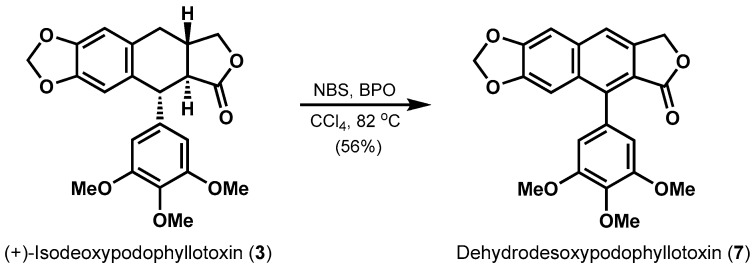
One-step conversion of tetralin to arylnaphthalene skeleton.

Next, the potential aromatization within tetralin lactone was investigated. As shown in Scheme 4, one-step conversion of (+)-isodeoxypodophyllotoxin (**3**) to dehydrodesoxypodophyllotoxin (**7**) was realized in 56% yield promoted by a mixture of *N*-bromosuccinimide (NBS) and dibenzoyl peroxide (BPO) in refluxing CCl_4_. The plausible mechanism of this tandem reaction would be radical bromination [26] catalyzed by BPO occurs firstly, and a fast elimination of the resulting labile benzylbromide followed by further oxidation, providing the central benzene ring in **7**. ^1^H NMR spectra data (Table S4) of synthetic dehydrodesoxypodophyllotoxin was consistent with previous report [27].

## 3. Materials and Methods

### 3.1. General Procedure

For product purification by flash column chromatography, SiliaFlash P60 (particle size: 40–63 μm, pore size 60A) and petroleum ether (bp. 60–90 °C) were used. All solvents were purified and dried by standard techniques and distilled prior to use. All of experiments were conducted under an argon or nitrogen atmosphere in oven-dried or flame-dried glassware with magnetic stirring, unless otherwise specified. Organic extracts were dried over Na_2_SO_4_ or MgSO_4_, unless otherwise noted. ^1^H and ^13^C-NMR spectra were taken on a Bruker AM-400, AM-600 and Varian mercury 300 MHz spectrometer with TMS as an internal standard and CDCl_3_ as solvent unless otherwise noted. HRMS were determined on a Bruker Daltonics APEXII 47e FT-ICR spectrometer with ESI positive ion mode. The X-ray diffraction studies were carried out on a Bruker SMART Apex CCD area detector diffractometer equipped with graphite-monochromated Cu-Kα radiation source. Melting points were measured on Kofler hot stage and are uncorrected.

### 3.2. Synthesis of C9a-PhSe-Deoxypicropodophyllin *(**4a*** and ***4b**)*

A solution of **2** [11] (100 mg, 0.25 mmol) in THF (8 mL) under argon was cooled to −78 °C, followed by the addition of freshly prepared LDA (0.5 mmol, 2.0 equiv). The stirred solution was maintained at this temperature for 20 min, and a solution of PhSeBr (118 mg, 0.5 mmol, 2.0 equiv) in THF (3 mL) was then added. The resulting mixture was stirred for 20 min at −78 °C, and then quenched by water (1 mL). The mixture was extracted with EtOAc (2 × 30 mL). The combined organic layers were washed with water (2 × 8 mL) and brine (8 mL) respectively, dried over Na_2_SO_4_, filtered and concentrated under reduced pressure. The crude product was purified by flash column chromatography (petroleum ether/EtOAc = 4:1 → petroleum ether/EtOAc =2:1) on silica gel to afford **4a** (90 mg, 65% yield) as a white solid and **4b** (42 mg, 30% yield) as a white solid. Characterization data for **4a**: *R_f_* = 0.42 (petroleum ether/EtOAc = 1:1); ^1^H-NMR (400 MHz, CDCl_3_): *δ* = 7.48 (d, *J* = 8.0 Hz, 1H), 7.47 (d, *J* = 8.0 Hz, 1H), 7.40 (t, *J* = 7.2 Hz, 1H), 7.28 (t, *J* = 7.2 Hz, 2H), 6.68 (s, 1H), 6.61 (s, 2H), 6.56 (s, 1H), 5.88 (d, *J* = 1.2 Hz, 1H), 5.87 (d, *J* = 1.2 Hz, 1H), 4.49 (s, 1H), 4.10 (dd, *J* = 9.2, 7.6 Hz, 1H), 3.85 (s, 3H), 3.84 (s, 6H), 3.75 (dd, *J* = 5.2, 4.0 Hz, 1H), 3.48 (dd, *J* = 16.4, 8.4 Hz, 1H), 3.32–3.27 (m, 1H), 2.62 (d, *J* = 16.4 Hz, 1H) ppm; ^13^C-NMR (100 MHz, CDCl_3_): *δ* = 176.7, 152.9 (2C), 147.2, 146.9, 137.7 (2C), 137.3, 134.6, 131.8, 129.9, 129.1 (2C), 126.1, 126.0, 109.3, 108.8, 106.8 (2C), 101.0, 73.3, 60.9, 56.2 (2C), 53.9, 51.3, 41.5, 35.0 ppm; HRMS (ESI): calcd. for C_28_H_30_NO_7_Se^+^ [M + NH_4_]^+^: 572.1182, found: 572.1186.

### 3.3. Synthesis of (+)-β-Apopicropodophyllin *(**5**)*

To a stirred solution of **4a** (90 mg, 0.076 mmol) in CH_2_Cl_2_ (4 mL) was added *m*-CPBA (77%, 34.0 mg, 0.15 mmol, 2.0 equiv) at 0 °C followed by the addition of NaHCO_3_ (12.6 mg, 0.15 mmol, 2.0 equiv). After stirring for 15 min, the reaction mixture was extracted with CH_2_Cl_2_ (3 × 20 mL). The combined organic layers were washed with saturated aqueous NaHCO_3_ (4 × 5 mL), water (5 mL) and brine (5 mL) respectively, then dried over Na_2_SO_4_, filtered and concentrated under reduced pressure. The resulting residue was purified by flash column chromatography (petroleum ether/EtOAc = 3:1 → petroleum ether/EtOAc = 1:1) on silica gel to afford (+)-β-apopicropodophyllin (**5**) (56 mg, 88% yield) as a white solid. *R_f_* = 0.37 (petroleum ether/EtOAc = 1:1); ${\left[\alpha \right]}_{D}^{20}$ = +92.04 (*c* = 1.00, CHCl_3_), ${\left[\alpha \right]}_{D}^{23}$ = +65.1 (*c* = 2.72, CHCl_3_)] [23]; m.p. 188–190 °C; ^1^H-NMR (300 MHz, CDCl_3_): *δ* = 6.72 (s, 1H), 6.63 (s, 1H), 6.37 (s, 2H), 5.954 (s, 1H), 5.947 (s, 1H), 4.90 (d, *J* = 17.4 Hz, 1H), 4.82 (d, *J* = 17.4 Hz, 1H), 4.81 (s, 1H), 3.86 (dd, *J* = 22.2, 3.9 Hz, 1H), 3.79 (s, 3H), 3.78 (s, 6H), 3.65 (dd, *J* = 22.2, 3.6 Hz, 1H) ppm; ^13^C-NMR (100 MHz, CDCl_3_): *δ* = 172.2, 157.2, 153.2 (2C), 147.3, 147.0, 138.3, 137.1, 129.7, 128.2, 123.8, 109.6, 107.7, 105.6 (2C), 101.3, 71.0, 60.8, 56.2 (2C), 42.8, 29.2 ppm.

This product (5 mg) was dissolved in EtOAc (1 mL) and hexane (2 mL). After three days, colorless single crystals were obtained by slow evaporation of solvents at room temperature.

### 3.4. Synthesis of (+)-γ-Apopicropodophyllin *(**6**)*

To a stirred solution of **4b** (42 mg, 0.16 mmol) in CH_2_Cl_2_ (3 mL) was added *m*-CPBA (77%, 72.0 mg, 0.32 mmol, 2.0 equiv) at 0 °C followed by the addition of NaHCO_3_ (26.9 mg, 0.32 mmol, 2.0 equiv). After stirring for 15 min, the reaction mixture was extracted with CH_2_Cl_2_ (3 × 20 mL). The combined organic layers were washed with saturated aqueous NaHCO_3_ (4 × 5 mL), water (5 mL) and brine (5 mL) respectively, then dried over Na_2_SO_4_, filtered and concentrated under reduced pressure. The resulting residue was purified by flash column chromatography (petroleum ether/EtOAc = 3:1 → petroleum ether/EtOAc = 1:1) on silica gel to afford (+)-γ-apopicropodophyllin (**6**) (26 mg, 88% yield) as a white solid. *R_f_* = 0.23 (petroleum ether/EtOAc = 1:1); ${\left[\alpha \right]}_{D}^{20}$ = +27.03 (*c* = 1.00, CHCl_3_), ${\left[\alpha \right]}_{D}^{19}$ = +25.0 (*c* = 1, CHCl_3_)] [28]; m.p. 206–208 °C; ^1^H-NMR (300 MHz, CDCl_3_): *δ* = 6.77 (s, 1H), 6.52 (brs, 3H), 5.97 (s, 2H), 4.70 (t, *J* = 8.7 Hz, 1H), 4.01 (t, *J* = 8.7 Hz, 1H), 3.92 (s, 3H), 3.83 (s, 6H), 3.39 (td, *J* = 15.9, 8.7 Hz, 1H), 2.94 (dd, *J* = 15.0, 6.9 Hz, 1H), 2.79 (dd, *J* = 15.6, 15.3 Hz, 1H) ppm; ^13^C-NMR (150 MHz, CDCl_3_): *δ* = 168.1, 152.7, 148.7 (2C), 147.3, 146.8, 138.1, 130.7 (2C), 129.9, 129.6, 119.9, 109.5, 108.6, 101.6 (2C), 70.9, 61.0, 56.2 (2C), 35.8, 33.3 ppm.

### 3.5. Synthesis of Dehydrodesoxypodophyllotoxin *(**7**)*

An oven-dried 10 mL round-bottom flask was charged with NBS (17.8 mg, 0.1 mmol, 1.0 equiv) and BPO (2.4 mg, 0.01 mmol, 0.1 equiv) at room temperature under argon, followed by the addition of a solution of **3** (40.0 mg, 0.1 mmol) in CCl_4_ (3 mL). The reaction mixture was stirred for 2 h at 82 °C. The reaction solvent was then evaporated in vacuo. The resulting residue was purified by flash column chromatography (petroleum ether/EtOAc = 5:1 → petroleum ether/EtOAc = 2:1) on silica gel to afford dehydrodesoxypodophyllotoxin (**7**) (22.2 mg, 56% yield) as a white solid. *R_f_* = 0.45 (petroleum ether/EtOAc = 1:1); m.p. 271–273 °C; ^1^H-NMR (400 MHz, CDCl_3_): *δ* = 7.70 (s, 1H), 7.21 (s, 1H), 7.12 (s, 1H), 6.55 (s, 2H), 6.09 (s, 2H), 5.38 (s, 2H), 3.97 (s, 3H), 3.84 (s, 6H) ppm; ^13^C-NMR (150 MHz, CDCl_3_): *δ* = 169.6, 153.0 (2C), 150.0, 148.7, 140.5, 139.8, 137.8, 134.6, 130.34, 130.30, 119.1, 118.7, 107.3 (2C), 103.8, 103.6, 101.8, 68.0, 61.0, 56.1 (2C) ppm.

## 4. Conclusions

In summary, a two-phase strategy was developed for the unified synthesis of (+)-β-apopicropodophyllin (**5**), (+)-γ-apopicropodophyllin (**6**), and dehydrodesoxypodophyllotoxin (**7**). In phase I, their tetrahydronaphthalene (THN) backbone was constructed by a Ni-catalyzed reductive cascade. In phase II, regioselective oxidation of stereodivergent tetralin lactone (**2** and **3**) gave arylnaphthalene lignan **7** and its dihydronaphthalene (DHN) congeners (**5** and **6**) efficiently.

## Supplementary Materials

The following are available online. Copies of ^1^H-, ^13^C-NMR, and crystallographic information files (CIFs) for **5**.
