# Supplementary material for: Concise Synthesis of (+)-β- and γ-Apopicropodophyllins, and Dehydrodesoxypodophyllotoxin
Source: Molecules. 2018 Nov 21;23(11):3037. doi: 10.3390/molecules23113037 (PMC6278515; doi:10.3390/molecules23113037)
Supplement: Supplementary file 1 [file molecules-23-03037-s001.pdf]

Supporting Information (experimental procedures and spectra data/copies) for

Concise Synthesis of (+)-*β*- and *γ*-Apopicropodophyllins, and Dehydrodesoxypodophyllotoxin

Jian Xiao [1,2], Guangming Nan [1], Ya-Wen Wang [2] and Yu Peng [2,3,*]

[1] University and College Key Lab of Natural Product Chemistry and Application in Xinjiang, Yili Normal University, Yining 835000, China; xiaoj2012@lzu.edu.cn (J.X.); nanguangming02@sohu.com (G.N.)
[2] School of Life Science and Engineering, Southwest Jiaotong University, Chengdu 610031, China; ywwang@swjtu.edu.cn
[3] State Key Laboratory of Applied Organic Chemistry and College of Chemistry and Chemical Engineering, Lanzhou University, Lanzhou 730000, China
* Correspondence: pengyu@lzu.edu.cn

Table of Contents

- X-ray Crystal Data of **5**•CH_2Cl_2 (*Table S1*)..S2
- ^{1}H NMR Data Comparison of *β*-Apopicropodophyllin (*Table S2*)...........................S3
- ^{1}H NMR Data Comparison of *γ*-Apopicropodophyllin (*Table S3*)…………….........S3
- ^{1}H NMR Data Comparison of Dehydrodesoxypodophyllotoxin (*Table S4*)………..S4
- ^{1}H NMR and ^{13}C NMR spectra of C9a-PhSe-Deoxypicropodophyllin (**4a**)………..S5
- ^{1}H NMR and ^{13}C NMR spectra of (+)-*β*-Apopicropodophyllin (**5**)..........................S7
- ^{1}H NMR and ^{13}C NMR spectra of (+)-*γ*-Apopicropodophyllin (**6**)...........................S9
- ^{1}H NMR and ^{13}C NMR spectra of Dehydrodesoxypodophyllotoxin (**7**).................S11

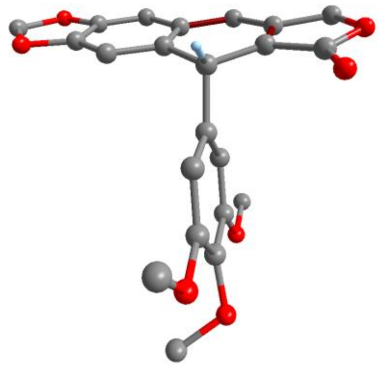

Table S1: X-ray crystal data of **5**•CH_2Cl_2

(selected H atoms and CH_2Cl_2 have been omitted for clarity)

Empirical formula	$C_{23}H_{22}O_7Cl_2$
Temperature (K)	297.16(10)
Crystal color	colorless
Formula weight	481.30
Crystal system	Monoclinic
Space group	$P2_1/c$
a (Å)	14.4878(12)
b (Å)	12.1979(8)
c (Å)	14.2602(12)
α (°)	90.00
β (°)	117.029(11)
γ (°)	90.00
V (Å ^{3})	2244.8(4)
Z	4
Density (calculated) (g/cm^3)	1.424
F (000)	1000.0
λ (Å)	1.54184
Reflections collected	8233
Independent reflections	4165
θ Range for data collection (°)	4.989-69.800
Index range	$-17 \leq h \leq 17$<br>$-14 \leq k \leq 7$<br>$-15 \leq l \leq 17$
Final R indices [I>2σ(I)]	R_1 = 0.0545, wR_2 = 0.1444
Largest difference peak and hole [e Å $^{-3}$]	0.367, –0.497

Table S2 ^{1}H NMR Data Comparison of *β*-Apopicropodophyllin

**5** (CDCl$_3$, 300 MHz)	ref.[1] (Toste *et al.*) (CDCl$_3$, 300 MHz)	
δ_H (ppm), *J* (Hz)	δ_H (ppm), *J* (Hz)	*Δδ* (ppm)
6.72 *s*	6.71 *s*	+0.01
6.63 *s*	6.62 *s*	+0.01
6.37 *s*	6.36 *s*	+0.01
5.954 *s*	5.94 *d* (1.2)	
5.947 *s*	5.93 *d* (1.2)	
4.90 *d* (17.4)		
4.82 *d* (17.4)	4.82 *m* (3H)	
4.81 *s*		
3.86 *dd* (22.2, 3.9)	3.85 *dd* (22.5, 4.5)	+0.01
3.79 *s*	3.78 *s*	+0.01
3.78 *s*	3.77 *s*	+0.01
3.65 *dd* (22.2, 3.6)	3.63 *dd* (22.5, 4.5)	+0.02

Table S3 ^{1}H NMR Data Comparison of *γ*-Apopicropodophyllin

**6** (CDCl$_3$, 300 MHz)	ref.[2] (Yamaguchi *et al.*) (CDCl$_3$, 300 MHz)	
δ_H (ppm), *J* (Hz)	δ_H (ppm), *J* (Hz)	*Δδ* (ppm)
6.77 *s*	6.77 *s*	0.00
6.52 br*s*	6.52 br*s*	0.00
5.97 *s*	5.97 *s*	0.00
4.70 *t* (8.7)	4.70 *dd* (8.8, 8.8)	0.00
4.01 *t* (8.7)	4.01 *dd* (8.8, 8.8)	0.00
3.92 *s*	3.92 *s*	0.00
3.83 *s*	3.84 *s*	–0.01
3.39 *td* (15.9, 8.7)	3.40 *m*	
2.94 *dd* (15.0, 6.9)	2.94 *dd* (16.0, 6.6)	0.00
2.79 *dd* (15.6, 15.3)	2.82 *d* (16.0)	–0.03

(1) Kennedy-Smith, J. J.; Young, L. A.; Toste, F. D. *Org. Lett.* **2004**, *6*, 1325.
(2) Kashima, T.; Tanoguchi, M.; Arimoto, M.; Yamaguchi, H. *Chem. Pharm. Bull.* **1991**, *39*, 192.

Table S4 ^{1}H NMR Data Comparison of Dehydrodesoxypodophyllotoxin

**7** (CDCl$_3$, 400 MHz)	ref.[3] (Tanabe *et al.*) (CDCl$_3$, 400 MHz)	
δ_H (ppm), *J* (Hz)	δ_H (ppm), *J* (Hz)	$\Delta\delta$ (ppm)
7.70 *s*	7.70 *s*	0.00
7.21 *s*	7.21 *s*	0.00
7.12 *s*	7.12 *s*	0.00
6.55 *s*	6.55 *s*	0.00
6.09 *s*	6.09 *s*	0.00
5.38 *s*	5.38 *s*	0.00
3.97 *s*	3.97 *s*	0.00
3.84 *s*	3.84 *s*	0.00

(3) Nishii, Y.; Yoshida, T.; Asano, H.; Wakasugi, K.; Morita, J.-i.; Aso, Y.; Yoshida, E.; Motoyoshiya, J.; Aoyama, H.; Tanabe, Y. *J. Org. Chem.* **2005**, *70*, 2667.

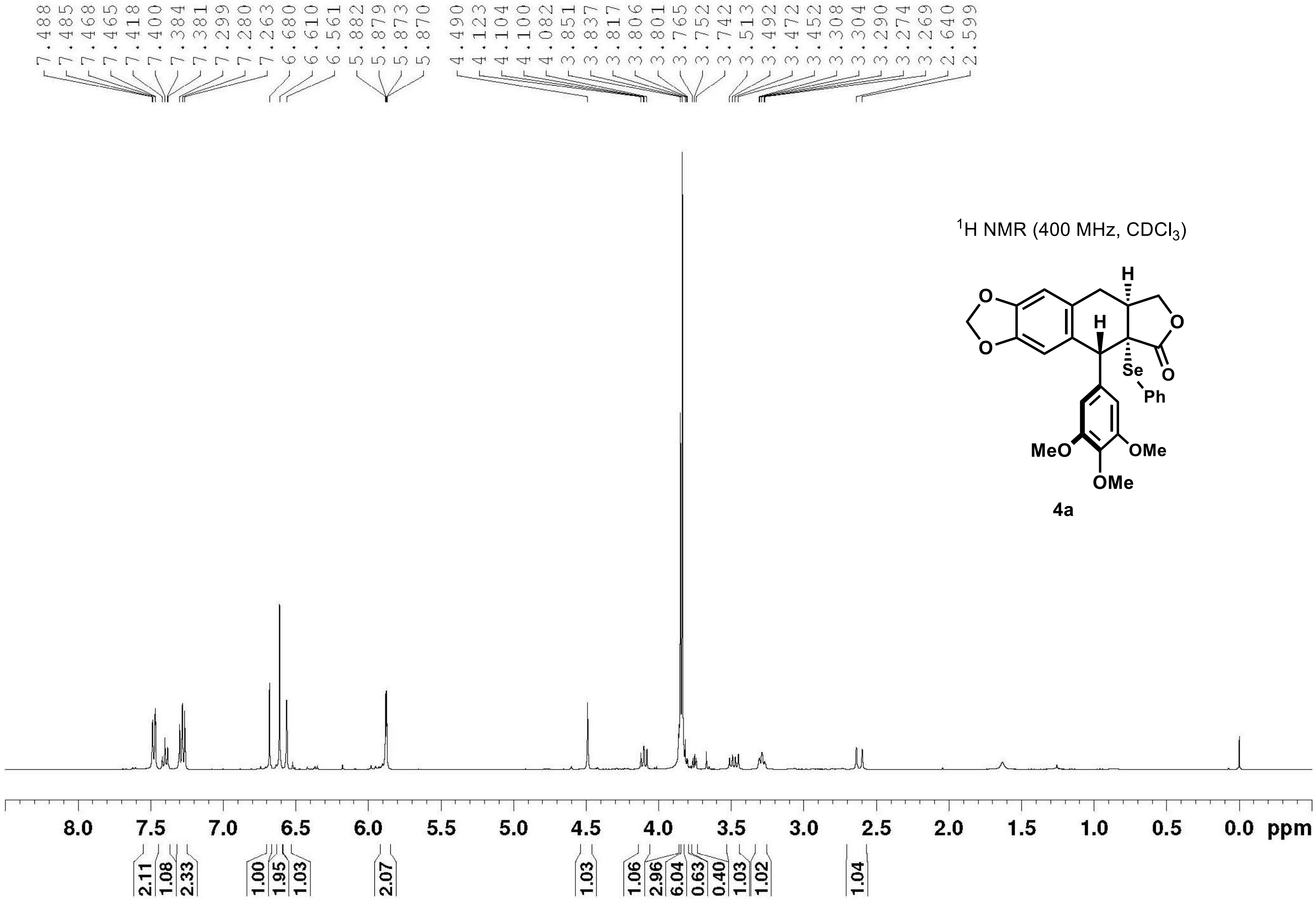

^{1}H NMR (400 MHz, $CDCl_3$)

4a

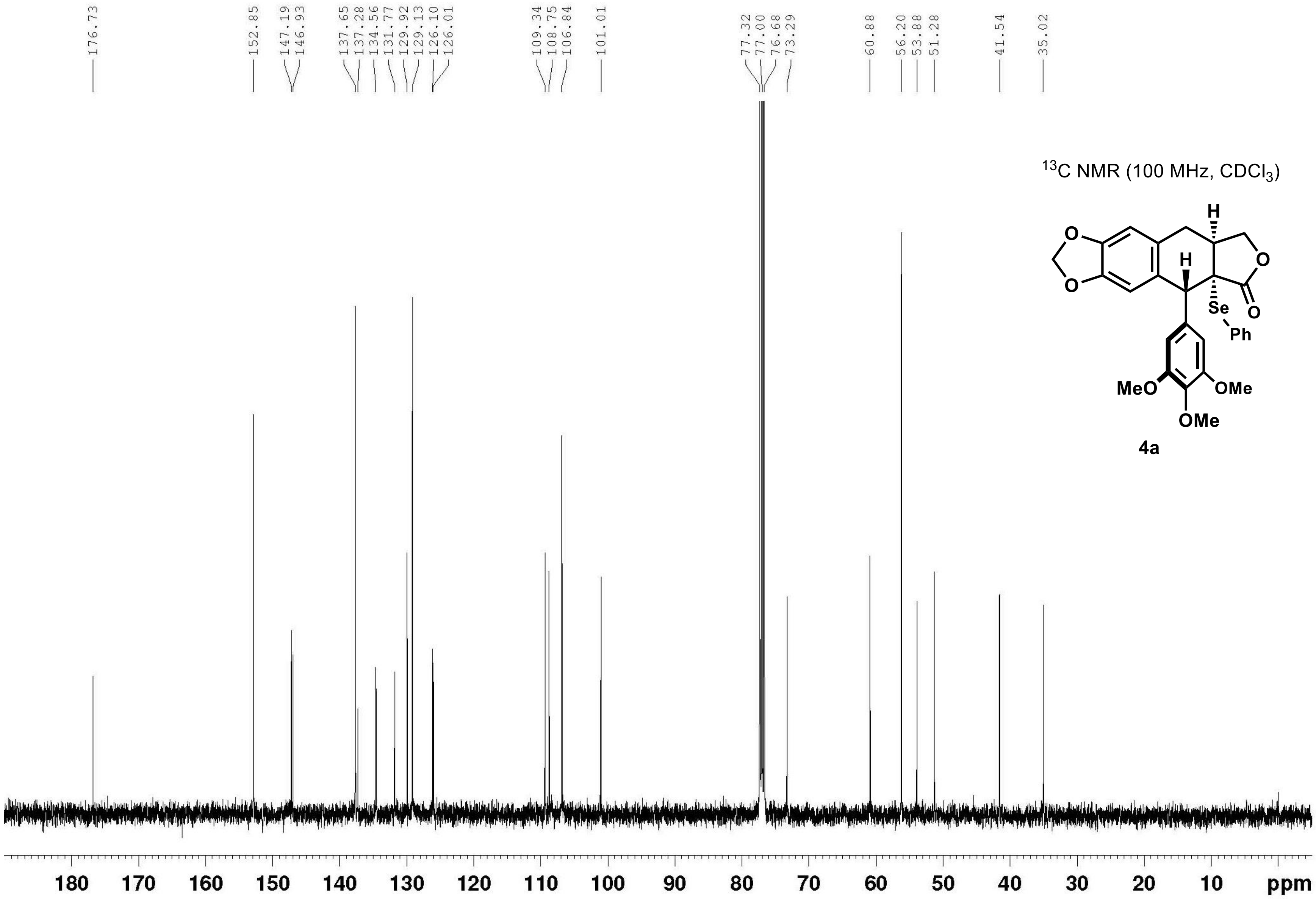

176.73
152.85
147.19
146.93
137.65
137.28
134.56
131.77
129.92
129.13
126.10
126.01
109.34
108.75
106.84
101.01
77.32
77.00
76.68
73.29
60.88
56.20
53.88
51.28
41.54
35.02
13C NMR (100 MHz, CDCl3)
H
O
O
H
O
Se
O
Ph
MeO
OMe
OMe
4a
180
170
160
150
140
130
120
110
100
90
80
70
60
50
40
30
20
10
ppm

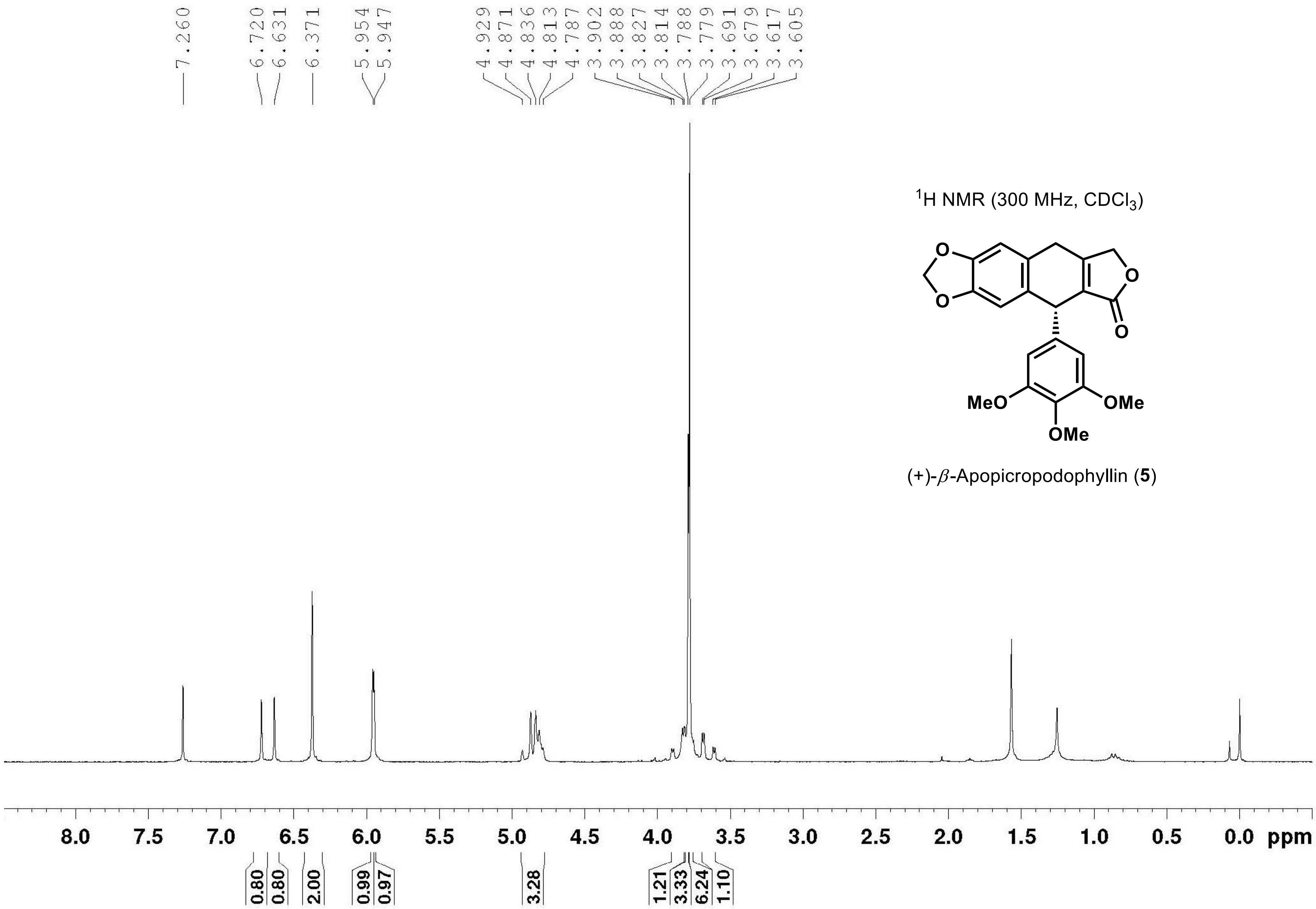
^1H NMR (300 MHz, $CDCl_3$)
O
O
O
O
MeO
OMe
OMe
(+)-β-Apopicropodophyllin (**5**)
7.260
6.720
6.631
6.371
5.954
5.947
4.929
4.871
4.836
4.813
4.787
3.902
3.888
3.827
3.814
3.788
3.779
3.691
3.679
3.617
3.605
8.0
7.5
7.0
6.5
6.0
5.5
5.0
4.5
4.0
3.5
3.0
2.5
2.0
1.5
1.0
0.5
0.0
ppm
0.80
0.80
2.00
0.99
0.97
3.28
1.21
3.33
6.24
1.10

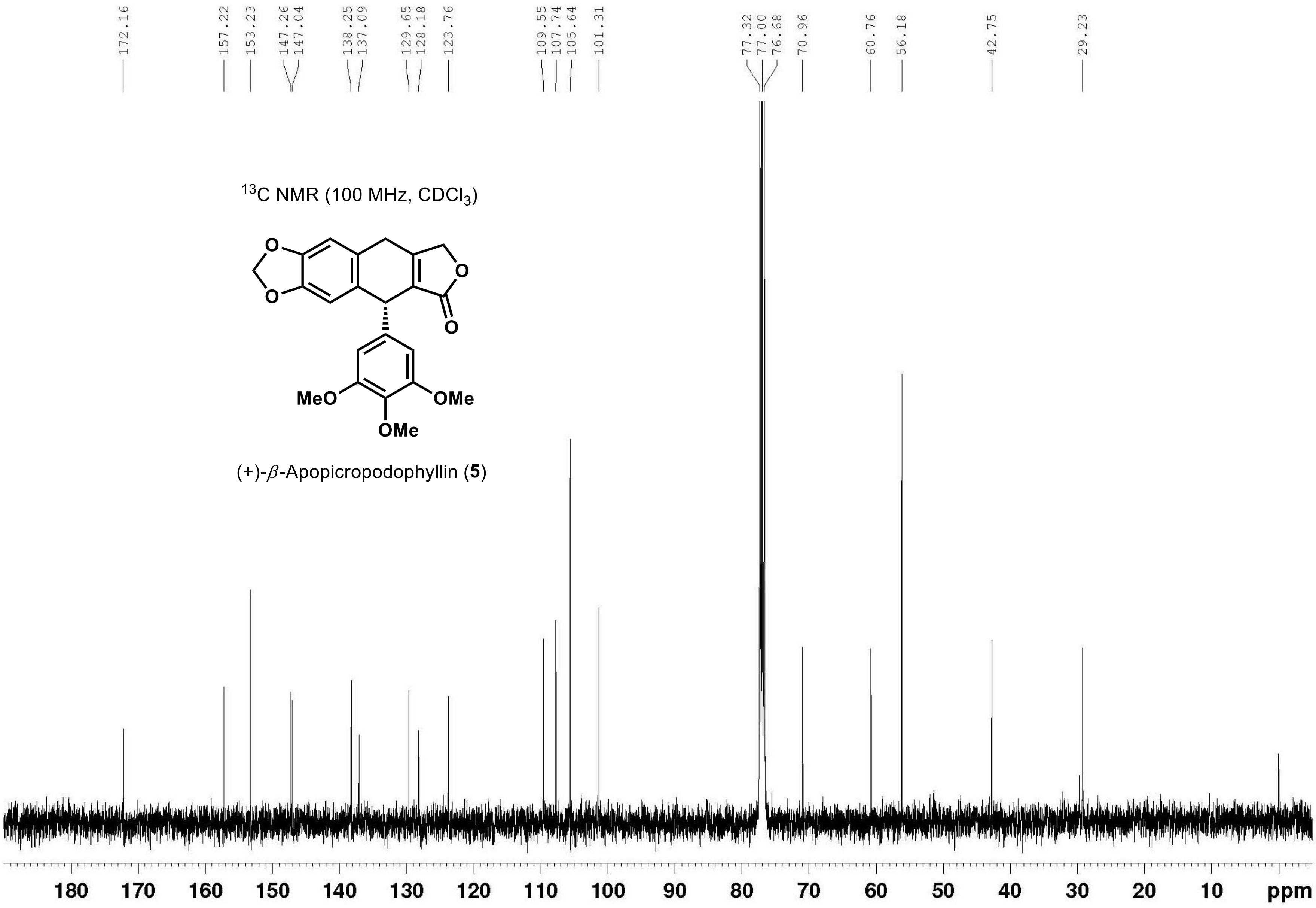

(+)-*β*-Apopicropodophyllin (**5**)

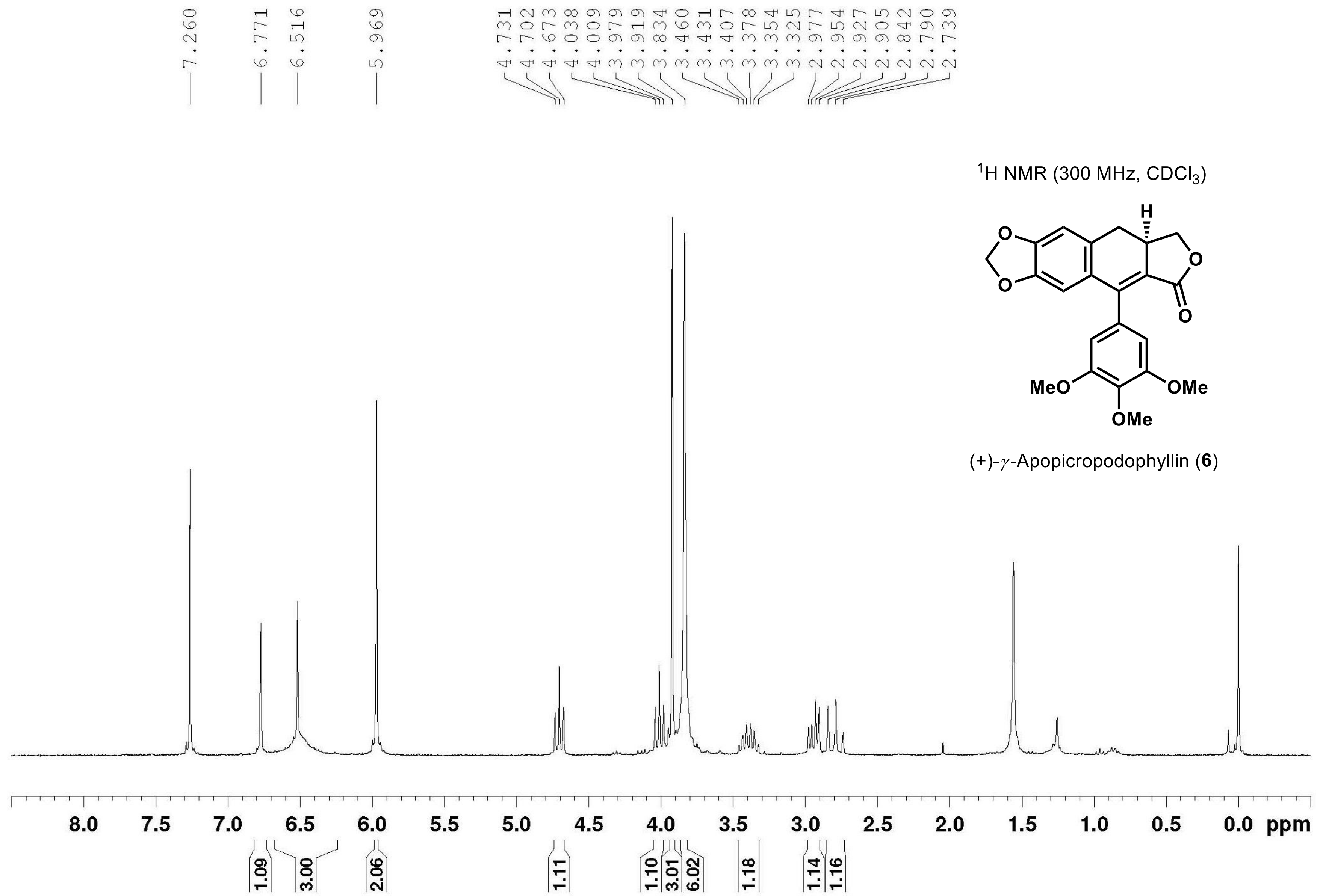

(+)-γ-Apopicropodophyllin (**6**)

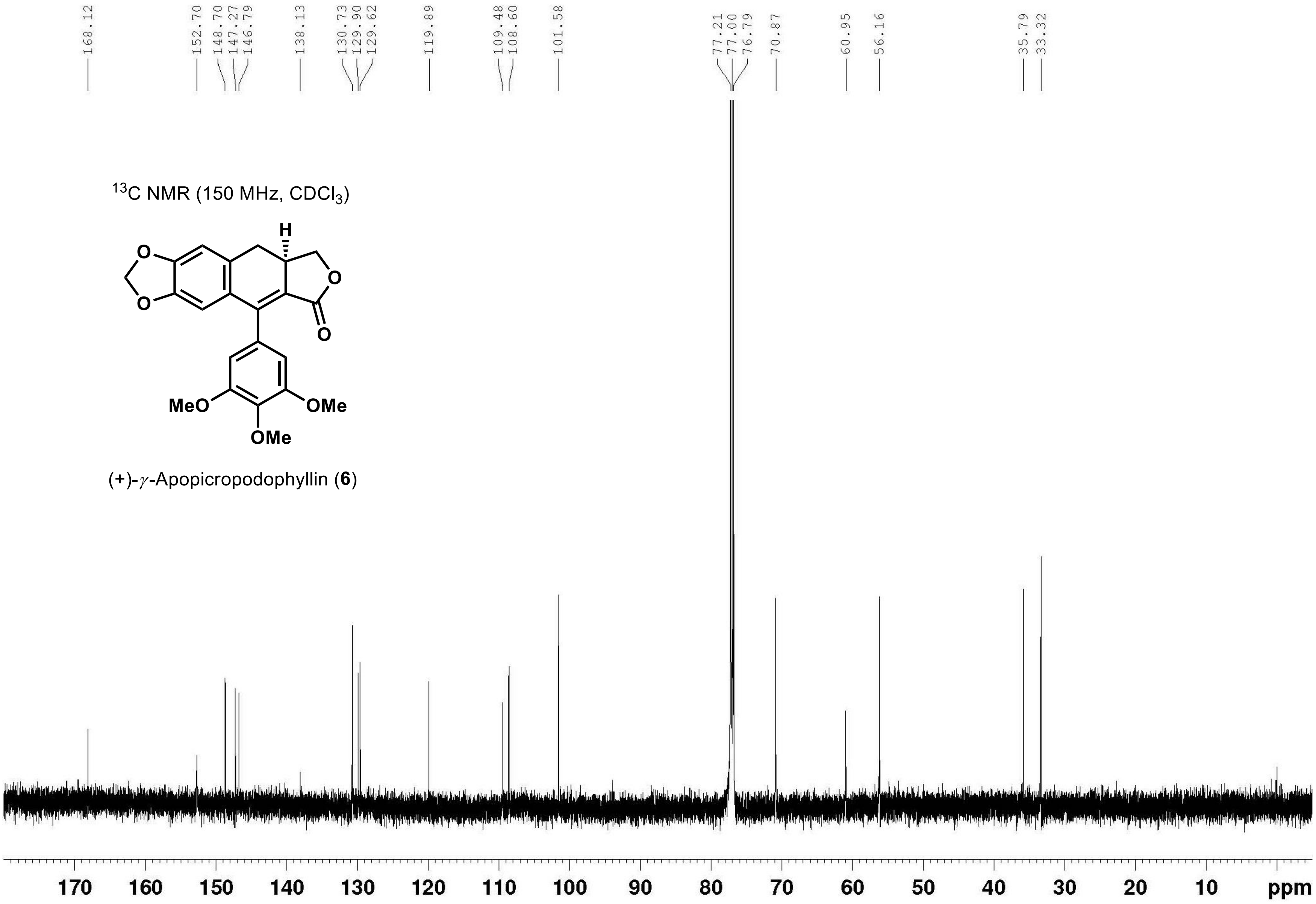

^{13}C NMR (150 MHz, $CDCl_3$)

(+)-γ-Apopicropodophyllin (**6**)

^{1}H NMR (400 MHz, $CDCl_3$)

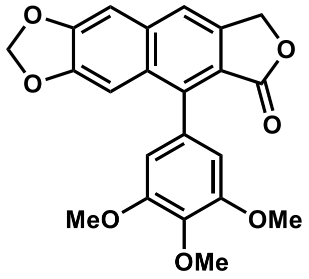

Dehydrodesoxypodophyllotoxin (**7**)

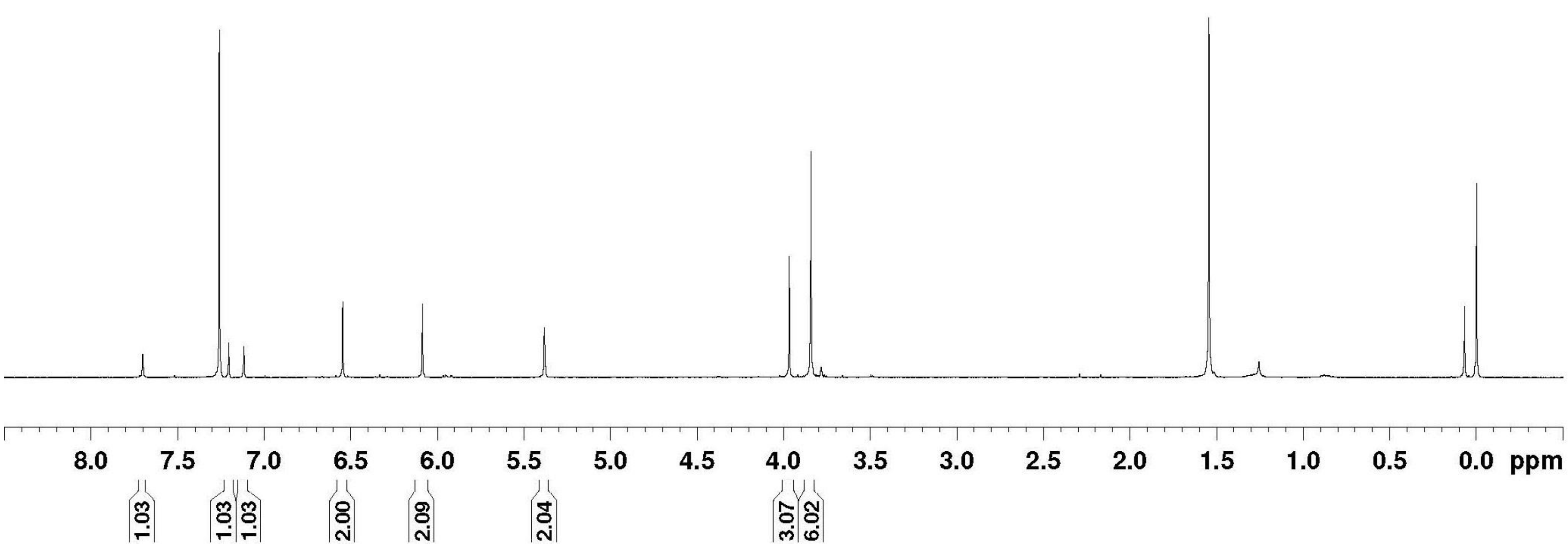

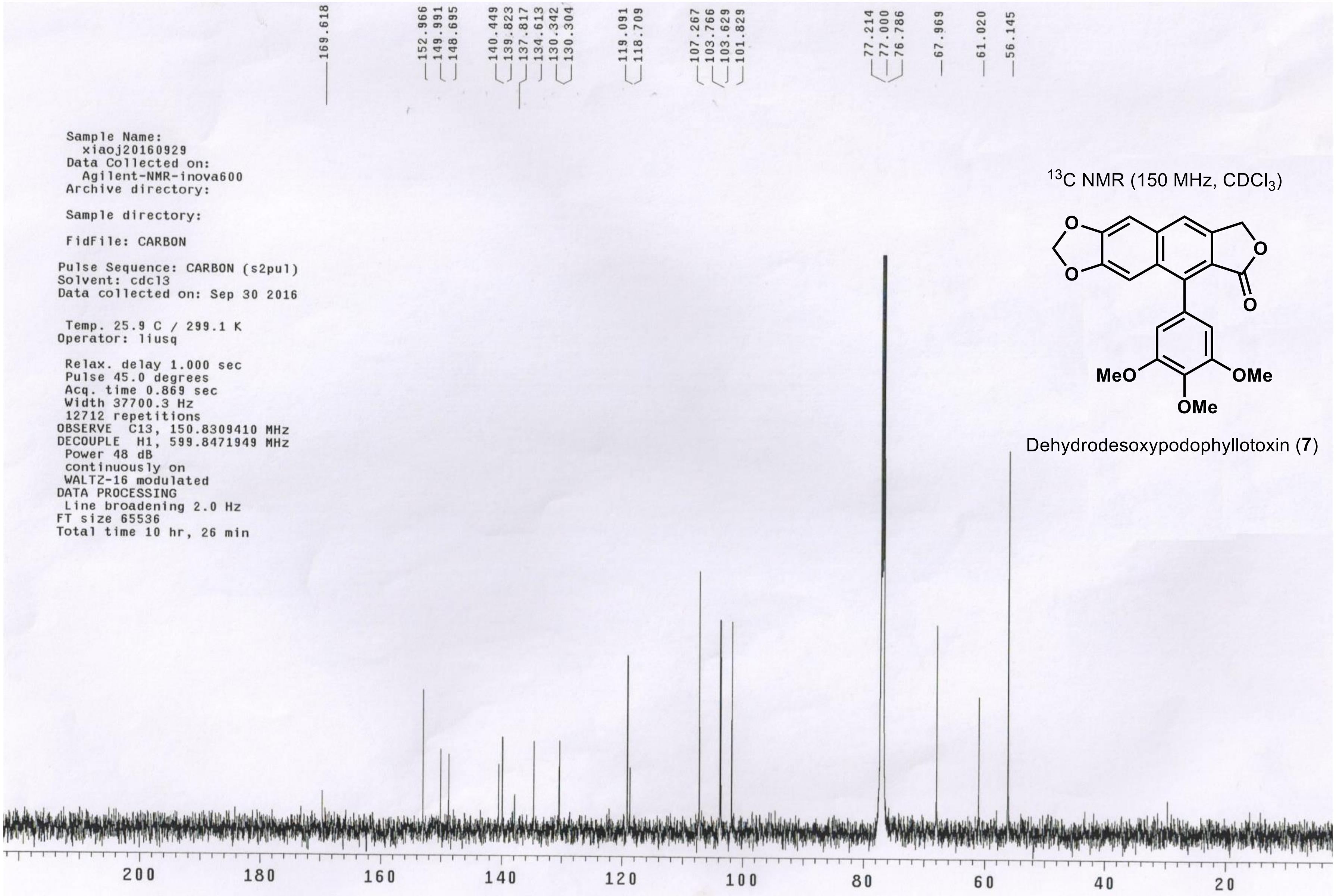

^{13}C NMR (150 MHz, $CDCl_3$)

Dehydrodesoxypodophyllotoxin (**7**)
